# Supplementary material for: Observational activation of anterior cingulate cortical neurons coordinates hippocampal replay in social learning
Source: bioRxiv. 2025 Aug 10:2024.03.31.587484. Originally published 2024 Apr 1. Preprint. [Version 2] doi: 10.1101/2024.03.31.587484 (PMC11014478; doi:10.1101/2024.03.31.587484)
Supplement: Supplement 1 [file NIHPP2024.03.31.587484v2-supplement-1.pdf]

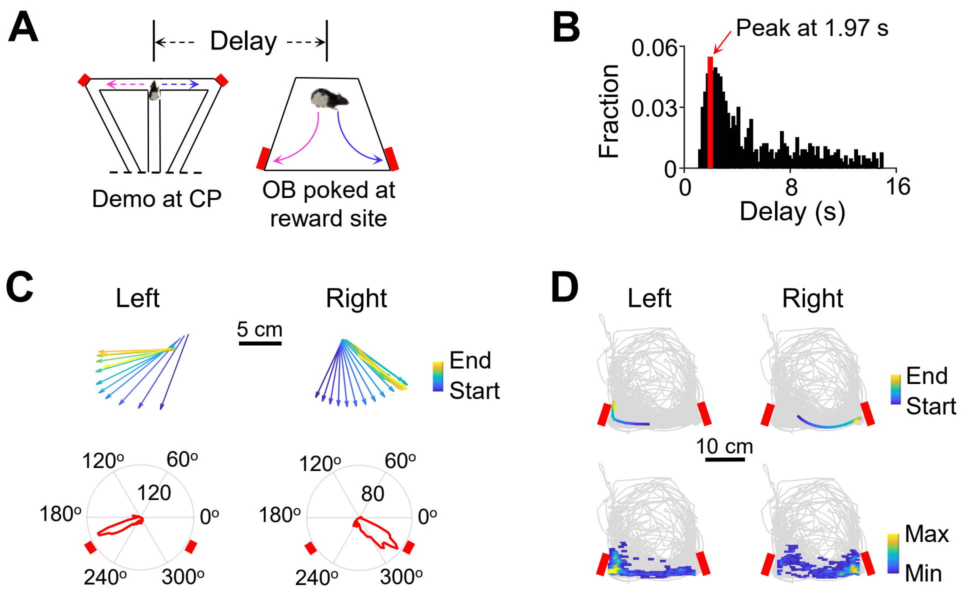

Figure S1. Behavior of OBs during delay periods in the observation box, related to Figure 2.

(**A**) Illustration of the delay time window of a trial: time interval between the Demo passing the T-maze choice point (CP) on a left (magenta) or right (blue) trajectory and the OB's first poke with reward at the corresponding water port in the box (not drawn proportionally).

(**B**) Distribution of the delay window duration for all trials of all recorded OBs under the Demo condition. Note the peak at 1.97 s.

(**C**) Top: an example OB's head direction within the 2 s (Start) delay period before first rewarded poke (End) in the box for a left and right trial. Head direction was computed in every 0.1 s time bin. Downward arrow shows the direction (270°) facing the T-maze.

Bottom: distribution of the OB's head direction in the 2 s delay periods of all trials in a session with left/right rewarded pokes. Number inside circle: radius length (count of time bins). Red rectangles: reward sites (at ~210°, 330°).

(**D**) Similar to (**C**), but for the OB's head position in the same left and right trial (top) and head position density during delay periods of all trials in the session. Bottom color bar: count of time bins. Gray trace: all head positions in the session.

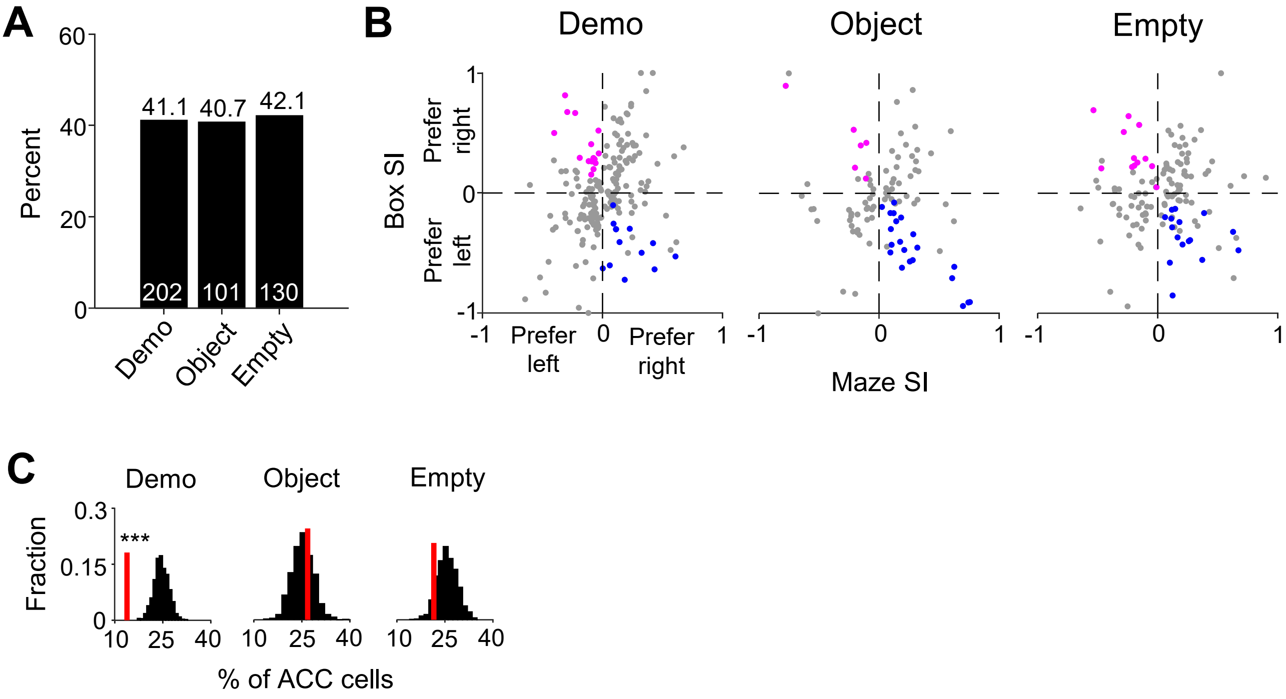

Figure S2. ACC cells with opposite-side selectivity in the box were fewer than the chance level under Demo, related to Figure 2.

(**A**) Numbers of ACC selective cells (white) and corresponding percentages among all recorded ACC cells under Demo, Object and Empty.

(**B**) SIs in the maze and in the box for all ACC selective cells under Demo, Object, and Empty. The plots are the same as in **Figure 2D**, but now the colored dots highlight those left- (magenta) or right- (blue) selective cells in the maze that had significant opposite-side selectivity in the box.

(**C**) Percentages of ACC selective cells with significant opposite-side selectivity in the box (red lines) under Demo, Object and Empty, compared to their random distributions (black) obtained by shuffling each cell's firing rates among all delay periods of left and right trials in the box. Demo: 14%, Z = -4.1, P = 2.1 x 10^{-5}, Z-test; Object: 27%, Z = 0.47, P = 0.32; Empty: 22%, Z = -1.2, P = 0.89.

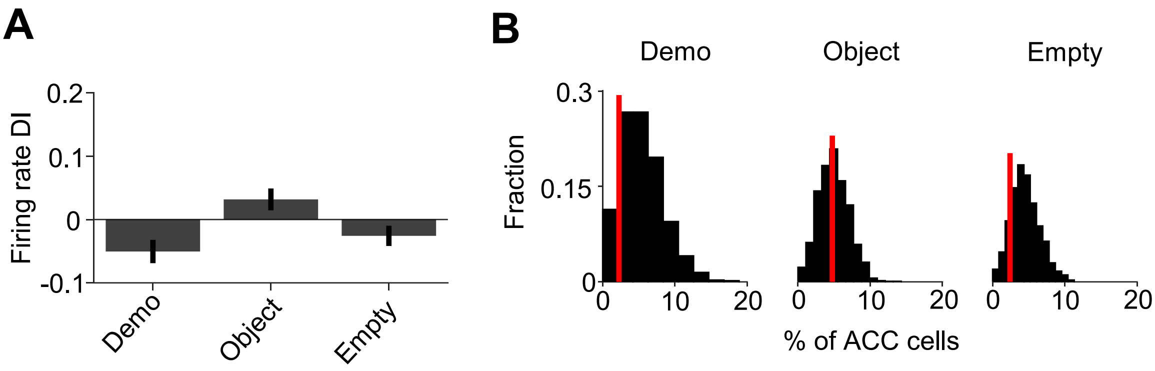

Figure S3. ACC cells with same-side selectivity in the box did not differ in firing rate during delay periods between correct and error trials on their non-preferred side, related to Figure 3.

(**A**) Average DIs (mean ± SEM) of ACC cells with same-side selectivity in the box on their non-selective side between delay periods of correct and error trials under Demo, Object and Empty. The DIs were not significantly different from 0 (Demo: -0.051 ± 0.030, N = 44, P = 0.11, two-sided t-test; Object: 0.032 ± 0.021, N = 84, P = 0.13; Empty: -0.026 ± 0.016, N = 124, P = 0.11). There was a significant difference among the 3 conditions (*One-way ANOVA*: $F_{(1,251)} = 3.54$, P = 0.030).

(**B**) Percentages of ACC cells with same-side selectivity in the box that had significantly higher rate during delay periods in correct trials on their non-preferred side (red, left-selective and right-selective cells combined) under Demo, Object and Empty, compared to shuffle-generated distributions (black). The percentage was not significant from the chance level under Demo (2.3%, Z = -0.77, P = 0.78, Z-test), Object (4.8%, Z = -0.0062, P = 0.50) or Empty (2.4%, Z = -1.2, P = 0.88).

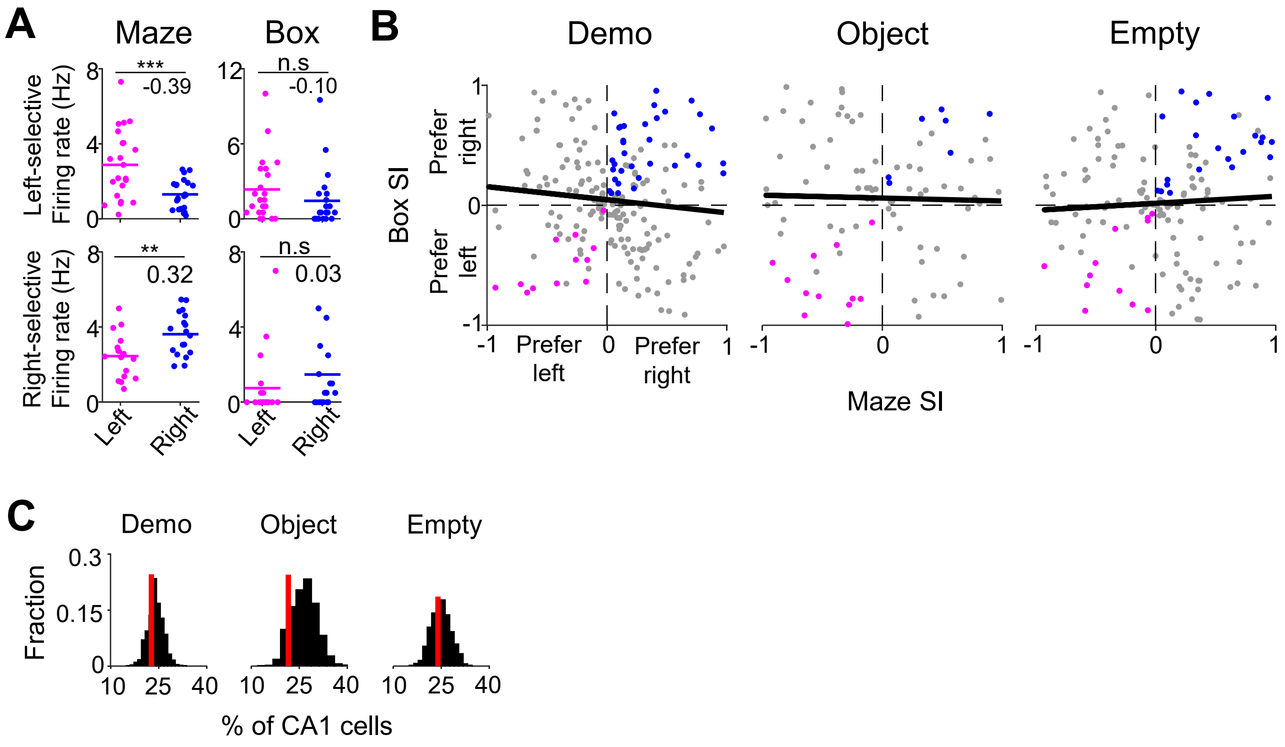

Figure S4. CA1 cells with same-side selectivity in the box were not significantly different from the chance level, related to Figure 4.

(**A**) Firing rates of two example CA1 cells during running in each trial on the left/right side of the maze and during each delay period in the box. Note different SIs (numbers) between the maze and box. (Left-selective cell: Maze: P = 3.3 x 10^{-4}, two-sided t-test; Box: P = 0.11; Right-selective cell: Maze: P = 0.0022; Box: P = 0.15)

(**B**) SIs in the maze and in the box for all CA1 cells active in the maze under Demo, Object and Empty. Colored dots: cells with significant same-side selectivity in the box on the left (magenta) or right (blue). Black line: linear regression between the maze and box SIs. There was no significant correlation under any of the conditions (Demo: R = -0.10, P = 0.93, *Pearson's r*; Object: R = -0.025, P = 0.59; Empty: R = 0.061, P = 0.24).

(**C**) Percentages of CA1 cells with significant same-side selectivity in the box (left/right combined, red lines) under Demo, Object and Empty, compared to their distributions (black) obtained by random shuffling of each cell's firing rates among all delay periods of left/right trials. The actual percentage was not significantly different from the random distribution under any of the conditions (Demo: 23%, Z = -0.54, P = 0.70, Z-test; Object: 22%, Z = -1.3, P = 0.91; Empty: 24%, Z = -0.38, P = 0.65).

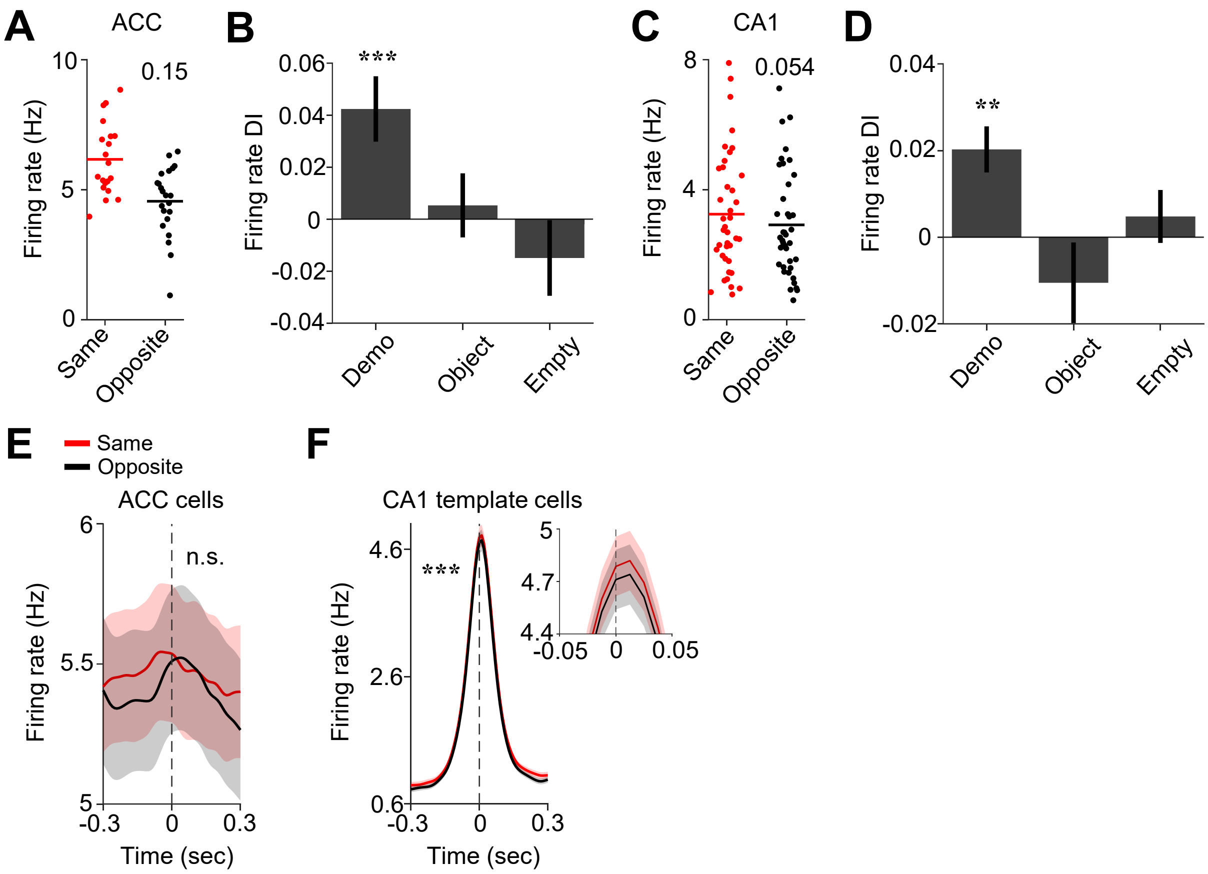

Figure S5. Activities of ACC selective cells and CA1 ensembles during water consumption in the box differed between same- and opposite-side of their selectivity, related to Figure 5.

(A) Trial-by-trial firing rates of a left-selective ACC cell during water consumption in the box on the same- and opposite-side of its selectivity in an example session. Each dot is a trial. Horizontal lines: mean values. Number: firing rate difference index (DI) between same- and opposite-side for this ACC cell.

(B) Average firing rate DIs over all ACC selective cells during water consumption under Demo, Object and Empty. The DIs were significantly higher than 0 under Demo (0.042 ± 0.013, N = 200, P = 9.1 x 10^{-4}, two-sided t-test), but not under Object (0.0053 ± 0.018, N = 99, P = 0.76) or Empty (-0.015 ± 0.018, N = 130, P = 0.41). There was a significant difference among the 3 conditions (*One-way ANOVA* test: $F_{(1,427)}$ = 4.0, P = 0.019).

(C-D) Similar to (**A-B**), but for firing rates of CA1 ensembles on the same- and opposite-side of ensembles. The average DIs in (**D**) were significantly higher than 0 under Demo (0.020 ± 0.0053, N = 19, P = 1.3 x 10^{-3}, , two-sided t-test), but not under Object (-0.011 ± 0.012, N = 11, P = 0.41) or Empty (0.0048 ± 0.0074, N = 13, P = 0.53). There was a significant difference among the 3 conditions (*One-way ANOVA* test: $F_{(1,42)}$ = 3.9, P = 0.028).

(**E**) Firing rate of ACC selective cells around SWRs on the same and opposite of their selectivity side in the box. Time bin: 0.01 s. Solid line: mean value. Shaded area: SEM. Dash line: peak time of SWR (time 0). There was no significant difference between Same and Opposite (*Two-way ANOVA* test: $F(_{1,60})$ = 2.1, P = 0.15).

(**F**) Same as (**E**), but for CA1 ensembles on the same and opposite of their selectivity. There was significant difference between Same and Opposite (*Two-way ANOVA* test: $F_{(1,60)}$ = 17.0, P = 3.8 x 10^{-5}). Insert: zoom-in around time 0 to show difference.

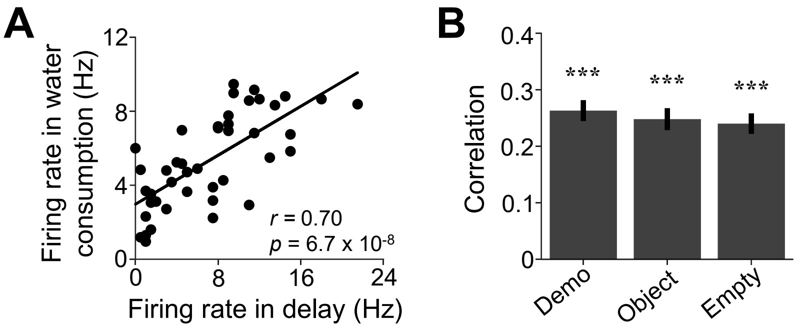

Figure S6. Activities of ACC selective cells during delay periods were correlated with their activities during water consumption periods in the box, related to Figure 6.

(**A**) Trial-by-trial correlation (*r*) between the firing rates of an example ACC cell during delay periods and during water consumption in all trials of a session. Each dot represents a trial.

(**B**) Average correlation values (mean ± SEM) for all ACC selective cells under Demo, Object and Empty. The correlations were significant under all the 3 conditions (Demo: 0.26 ± 0.019, N = 200, P = 5.7 x 10^{-32}, two-sided *t*-test compared to 0; Object: 0.25± 0.028, N = 99, P = 2.1 x 10^{-14}; Empty: 0.24 ± 0.023, N = 130, P = 2.1 x 10^{-19}). There was no significant difference among the 3 conditions (*One-way ANOVA*: $F_{(1,428)}$ = 0.32, P = 0.73). The result indicates that the high (or low) rate of an ACC selective cell during the delay period in a trial was accompanied with its high (or low) rate during the water consumption of the same trial.
